# Conservative treatment for osteoid osteoma of the odontoid process of the axis: a case report

**DOI:** 10.1186/1477-7819-12-305

**Published:** 2014-10-06

**Authors:** Jun Qiao, Feng Zhu, Zezhang Zhu, Zhen Liu, Bangping Qian, Yong Qiu

**Affiliations:** Spine Surgery, Drum Tower Hospital, Nanjing University Medical School, 321 Zhongshan Road, Nanjing, 210008 China

**Keywords:** osteoid osteoma, axis, cervical, spine

## Abstract

**Background:**

Osteoid osteoma is a primary benign bone lesion, which constitutes about 10% of all primary benign bone tumors and 3% of all primary bone tumors. The spine is involved in 10% of the cases, and the lumbar spine is the most commonly affected whereas the tumor is rarely seen in the cervical spine. With regard to the osteoid osteoma being located at the odontoid process of the axis, limited cases have been reported in the literature.

**Case presentation:**

An osteoid osteoma of the odontoid process of the axis was diagnosed by computed tomography in an 18-year-old male patient with a 3-month history of pain. The patient’s parents refused surgery for fear of surgical risks and high expense. Considering the benign nature of osteoid osteoma, we prescribed celecoxib 200 mg per day to the patient. With the treatment, the patient’s pain was alleviated gradually and the range of motion of the cervical spine also recovered to normal. At the two-year phone follow-up, the patient was free of symptoms.

**Conclusions:**

For this kind of benign tumor, conservative treatment plus close follow-up is applicable whereas surgery bears significant risks and a heavy economic burden.

## Background

Osteoid osteoma, a primary benign bone lesion, was first defined by Jaffe in 1935 [1]. It pathologically features a highly vascularized nidus of connective tissue surrounded by sclerotic bone [2, 3]. The nidus measures about 10 mm in diameter, and the size is the main distinguishing feature between it and osteoblastoma [4]. Osteoid osteoma constitutes about 10% of all primary benign bone tumors and 3% of all primary bone tumors [5]. Most of the cases occur in the first three decades and occurs two to three times more frequently in men than in women [6]. Predilection sites of the osteoid osteoma are long bones, especially those of the lower extremities. The spine is involved in 10% of the cases, and the lumbar spine is the most commonly affected whereas the tumor is rarely seen in cervical spine [7]. With regard to the osteoid osteoma being located at the odontoid process of the axis, limited cases have been reported in the literature [8, 9]. Herein, we report an osteoid osteoma of the dens axis confirmed by radiographic examinations.

## Case presentation

**Figure 1:**
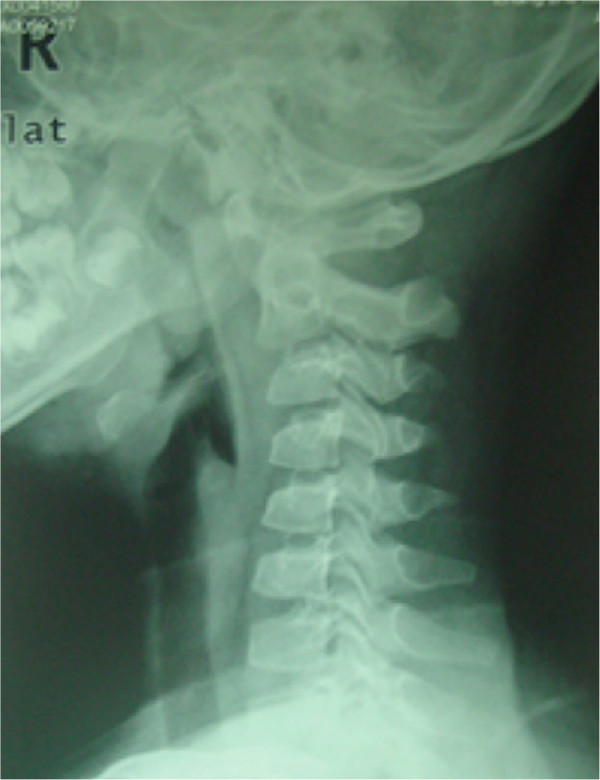
**Lateral X-ray showing kyphosis of cervical spine.**

**Figure 2:**
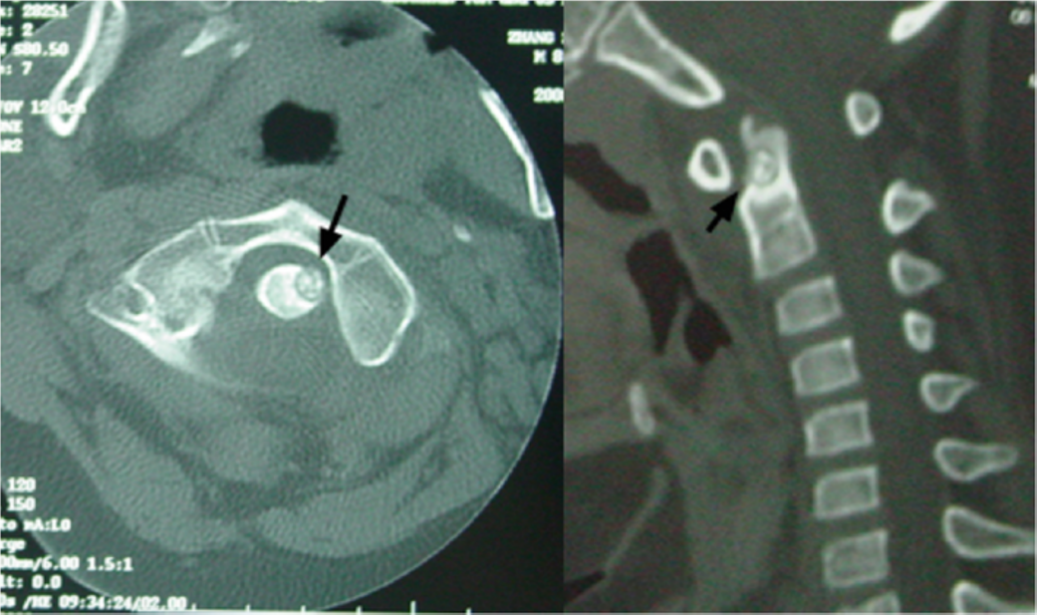
**Transversal computed tomography (CT) images showing the nidus with partial ossification of matrix and sclerotic margin, and sagittal CT images of the nidus.**

**Figure 3:**
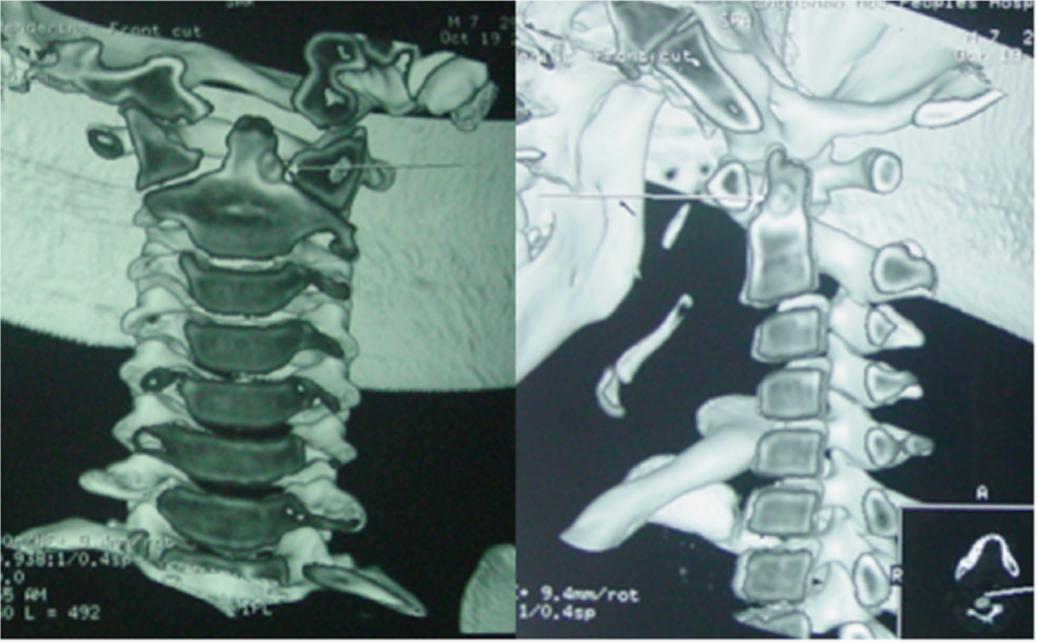
**Computed tomography (CT) of three-dimensional (3-D) reconstruction of the nidus.**

An 18-year-old male patient had a history of neck pain of 3 months’ duration, which was more severe at night. A physical examination revealed a remarkably reduced rotation to the right and mild kyphosis. A lateral cervical spine radiograph showed kyphosis of the cervical spine (Figure 1). A computed tomography (CT) scan revealed a lytic area involving the odontoid process of the axis with the partial ossification of the matrix and a sclerotic margin (Figure 2). By CT three-dimensional (3-D) reconstruction, the nidus was found at the conjunction of the odontoid process of the axis and the body of the axis (Figure 3).

From the patient’s medical history and radiological and physical examination, we made a diagnosis of osteoid osteoma of the odontoid process of the axis. The patient’s parents refused surgery for fear of surgical risks and high expense. Considering the benign nature of osteoid osteoma, we prescribed celecoxib 200 mg per day to the patient. With the treatment, the patient’s pain was alleviated gradually, and the range of motion of the cervical spine also recovered to normal. At the two-year phone follow-up, the patient was free of symptoms. However, he refused to go to our outpatient center for radiographic examinations.

## Discussion

Osteoid osteoma is a benign tumor, for which the chief symptom is pain whereas other clinical symptoms are related to its location [10]. The pain is localized and may be aggravated with motion. At the same time, pain can also be alleviated with activity [11]. Localized pain is caused by the nerve fibers in the nidus. The production of prostaglandin may lead to an increase in vascular pressure, which may produce pain by stimulating afferent nerves around the nidus [12]. The pain is more severe at night and is relieved by non-steroidal anti-inflammatory drugs (NSAIDs) [13]. This feature could also be used as a diagnostic clue. Scoliosis is reported in 70% of the cases when the lesion involves the spine, and it is the most common cause of painful scoliosis in adolescents, especially when the lesion is located in lumbar or thoracic spine [14–16].

CT is recommended as the best diagnostic tool to define and localize the nidus, particularly when the nidus is located at the spine [13]. With CT scanning, we can optimally visualize the nidus with perifocal marginal sclerosis [17]. Conventional radiography is insufficient to visualize the lesion because of the complicated anatomy of the spine [17]. Magnetic resonance imaging (MRI) is not as accurate as CT in demonstrating the nidus because the nidus presents different signal intensity in different patients [18–20]. The increased signal intensity of the lesion on T2-weighted images or on enhanced T1- weighted images was pathologically correlated with the degree of vascularity of the fibrovascular nidal stroma and the amount of osteoid substance within the nidus [21]. On the other hand, for detecting changes in soft tissue and bone marrow around the nidus, MRI is more sensitive than CT. These changes are due to bone marrow inflammation and edema [18, 21]. In addition to CT and MRI, bone scintigraphy is also a sensitive diagnostic test.

Tumors that affect the axis vertebra are numerous and include osteoblastoma, eosinophilic granuloma, chondroma, paraganglioma, plasmacytoma, multiple myeloma and so on [22]. Osteoblastoma is the major differential diagnosis of osteoid osteoma because they have the same pathological features but distinct natures. In contrast to osteoid osteoma, osteoblastoma is more aggressive, often extending to extraskeletal soft tissues. Moreover, it often recurs and even metastasizes after surgery [23–25]. In pathology, the two tumors are both lesions of osteoblastic origin [7, 26]. The most significant difference between them is the size of the nidus. A lesion is diagnosed as osteoid osteoma when its diameter is less than 15 mm and as osteoblastoma when larger [27].

Surgeons often hold progressive attitudes toward the treatment of osteoid osteoma. For example, *en block* excision is frequently recommended [28, 29]. In addition, minimally invasive methods, such as CT-guided thermocoagulation and percutaneous radiofrequecy ablation have also obtained satisfactory outcomes [30–32]. When the tumor is combined with scoliosis, surgical excision with or without correction surgery is widely adopted. However, surgical treatment is by no means the only choice, and surgeons should weigh costs against benefits before a decision is made. In the present case, conservative treatment was administered in consideration of economic issues and surgical risks. In developing countries, the coverage of medical insurance is inadequate, and many patients cannot afford surgical expenses. For this kind of benign tumor, conservative treatment plus close follow-up is applicable. Moreover, the risky anatomic location of the lesion and the high morbidity further prompted us to choose medication, with a satisfactory outcome [13, 33].

## Conclusions

For this kind of benign tumor, conservative treatment plus close follow-up is applicable whereas surgery bears significant risks and a heavy economic burden.

## Consent

Written informed consent was obtained from all patients enrolled in the investigation. The study protocol conformed to the ethical guidelines of the 1975 Declaration of Helsinki and the guidelines of the regional ethical committees of Zurich, Switzerland, and Basel, Switzerland.

## Acknowledgements

This work was supported by National Key Clinical Department Project and Talents Programme of Jiangsu (Grant No. WSW-005). We thank MS Zhang Linlin for her contribution to this article.

## Abbreviations

CT: computed tomography
MRI: magnetic resonance imaging
NSAIDs: non-steroidal anti-inflammatory drugs.
